# 

**Published:** 1829-08

**Authors:** 


### MONTHLY LIST OF MEDICAL BOOKS.

[*Medical Works cannot be entered on this List except a copy be sent for the purpose; the titles of Books having frequently been transmitted to us, as published, which have not appeared for weeks, or even months, after.*]

An Experimental Inquiry into the Laws which regulate the Phenomena of Organic and Animal Life. By GEORGE CALVERT HOLLAND, M.D. &c. &c. —8vo. pp. 462. Edinburgh and London, 1829.

An Essay on the Connexion between the Action of the Heart and Arteries and the Functions of the Nervous System, and particularly its influence in exciting the involuntary Act of Respiration. By JOSEPH SWAN.—8vo. pp. 162. Longman, 1829.

An Essay upon the Treatment of the Deep and Excavated Ulcer: with Cases. By RICHARD ANTHONY STAFFORD, M.R.C.S. and lately House-Surgeon to St. Bartholomew's Hospital.—8vo. pp. 72. 1829.

Medical Botany, Nos. XXX. and XXXI. By JOHN STEPHENSON, M.D. F.L.S. and JAMES MORSS CHURCHILL, F.L.S. Lecturer on Midwifery, &c. —Published by TILT, 86, Fleet street.

The graphical embellishments of these Numbers are quite equal to the preceding, and the descriptive text as practically instructive.

Essay on a Morbid Affection of Infancy, arising from circumstances of Exhaustion, but resembling Hydrencephalus. By MARSHALL HALL, M.D. F.R.S.E.—Pp. 40. 1829.

Address of Earl STANHOPE, President of the Medico-Botanical Society, at the Anniversary Meeting, January 1829.

An Oration delivered before the Medico-Botanical Society of London, at the commencement of its ninth Session. By JOHN FROST, F.R.S. Edin. F.L.S. Director of the Medico-Botanical Society of London. Dedicated, by permission, to the King.—Treuttel and Würtz, 1828.

A Practical Synopsis of Cutaneous Diseases, according to the arrangement of Dr. WILLAN: exhibiting a concise View of the Diagnostic Symptoms and the Method of Treatment. By THOMAS BATEMAN, M.D. F.L.S. &c. The Seventh Edition. Edited by A. T. THOMSON, M.D. F.L.S. Member of the Royal College of Physicians, and Professor of Materia Medica and Pharmacy in the University of London, &c.—8vo. pp. 460. Longman, 1829.

### NOTICES.

*Communications have been received from* Mr. CHENEVIX, Mr. PAXTON, Mr. HODSON, &c.

MEDICUS" *attaches too much importance to the scribbler he mentions.*

